# On the Process of Becoming a Great Scientist

**DOI:** 10.1371/journal.pcbi.0040033

**Published:** 2008-02-15

**Authors:** Morgan C Giddings

In the Editorial “Ten Simple Rules for Doing Your Best Research, According to Hamming” [1], Erren and colleagues discussed ten ideas originally presented by Hamming for how to do great science. I am grateful that the authors started this discussion. Scientific careers are very challenging, and there is a lack of training in many graduate programs to provide this kind of career meta-advice. Such discussions are a good starting point, and young scientists should take them seriously.

In the vein of promoting further debate and discussion, I provide here a different and perhaps deeper look at what makes a successful scientist. While I can't claim to have the reputation of Hamming, I grew up in a family of well-known scientists, and have had plenty of chances to observe the trajectories of scientific careers over my lifetime. Based on that experience, I propose the following as a somewhat distinct set of guidelines for doing the best research:

## 

### 1. Don't worry about age, worry about being exposed to new ideas.

While it appears that age plays a role in scientific creativity, it has not been well examined whether that role is biologically causative. There are many *social* changes that usually occur as anyone ages, which may play a greater role than biology does in the age-related creativity decline. Older scientists usually become boxed into their fields of expertise, and come to be seen as “experts.” As such, they are less likely to have their ideas directly challenged by others, and less likely to be exposed to radically new ideas or different fields. I have seen many anecdotal references to Einstein's creative powers reducing as he aged, as his best work was done in his 20s. But this ignores a major factor: during his creative years, he was a patent clerk who was seen as a “nobody,” whereas in his later years he was an eminent professor. Being a nobody has certain creative advantages—for one, there is not much to lose by promoting radical new ideas, because one has no reputation or established career at stake. Also, one is not expected to follow the “party line,” regardless of the latest scientific fashion that happens to be in vogue.

Promoting new ideas can often be a minefield for one's career, since there is usually a long period of violent resistance to new ideas. Barry Marshall had to drink a culture of H. pylori to give himself an ulcer, in order to overcome resistance to the idea that this organism caused ulcers [2]. Now, more than 20 years later, he and co-discoverer Robin Warren have the Nobel Prize, and the role of H. pylori in ulcers is widely accepted.

In today's competitive grant world, this phenomenon is exacerbated. It is dangerous to one's funding to go against the trend, and if there is a lab to support and mouths to feed, the disincentives are great. This phenomenon stifles creativity, perhaps far more than biological age does.

If one is therefore concerned about retaining scientific creativity, perhaps the best solution is to force exposure to new ideas, concepts, and people. Hamming also discussed the importance of this kind of exposure by “keeping your door open” [3]. I think that more than just keeping one's door open, a more direct way of doing this is to become involved in entirely new fields from time to time, which tends to promote creative thinking outside established dogma. So, don't worry about your age, worry about whether you are continuing to expose yourself to new and challenging ideas.

### 2. Tinker.

While it is not frequently acknowledged either in the popular press or in scientific literature, a significant fraction of scientific discovery is the result of serendipity (or to put it more bluntly, luck). From the discovery of penicillin by Fleming to the discovery of new ionization techniques such as MALDI that power modern mass-spectrometry based proteomic research, luck has frequently played a big role. Such discoveries are generally attributed to hard work and genius, rather than to luck. Doing so gives the “genius” too much credit and luck too little.

Often the big discoveries come from someone noticing an inconsistency or oddity in their surroundings or experiments, then doggedly working to figure out what is causing it. So perhaps being a great scientist is less about “genius” than it is about willingness to pursue the unusual at the expense of pursuing the usual. This comes back to the argument about age: often, once one has become entrenched in a paradigm, blindness to inconsistencies grows, and so it takes someone from outside of a field to point those out and pursue them.

This should be encouraging news for those of us who don't consider ourselves geniuses. The best way to promote scientific success may be to maximize exposure to chance occurrence and events—especially those that have more upside than downside potential. So, don't just ignore those little inconsistencies that arise in your work, give them some room for consideration. This is something anyone can do, though it takes time and courage (see point 3, below).

In addition, to be creative and remain open to fortuitous occurrences, the mind needs a rest from time to time. One can be buried in the lab 20 hours a day, and easily become lost in the self-created world where the little oddities begin to escape notice. Fleming discovered penicillin upon return from a long vacation, and his fresh mind may have contributed to the key observation he made on the effect of mold upon bacterial cultures. So it is critical to balance hard work with other activities, particularly those that provide exposure to new and different challenges: travel, sports, hobbies, family, or whatever.

### 3. Take risks.

Risk taking is where most of the big discoveries in science lie. Recall Dr. Marshall and H. pylori: he was willing to swallow a culture of the bacterium to prove his theory. And later, he shared the Nobel Prize for it. It may not be wise to go around drinking random bacterial cultures in the hopes of discovering something new. But it is important when something outside the current scientific fashion is discovered, to at least consider the risks and possible payoffs of pursuing it. Those who do pursue such ideas may find it hard to get funding for them. Others may say it is a bad idea. People may reject papers, expressing vehement opposition to a new idea. For really groundbreaking ideas, there may even be hecklers at talks! But, as Hamming pointed out in his lecture: “The great scientists, when an opportunity opens up, get after it and they pursue it” [3].

Pursuing new lines of inquiry can be very discouraging at times, but it is all part of the process any new idea goes through to transform from fringe to mainstream. I recall one major experience I had with this. Around 1996, I came up with an idea for doing DNA sequencing reactions in a test tube in a way that is very much like pyrosequencing today. After presenting it to a mentor and having it shot down, I gave up on it and went back to my “safe” work. While that was not a great time to pursue a new line of work outside my graduate studies, perhaps I should not have given up so quickly, considering the importance of pyrosequencers now.

Risk taking may be a particular challenge for female scientists. It seems that cultural norms discourage risk taking in young girls more so than in boys, and this can carry forward through to adulthood and into scientific careers. The top female scientists I know of take risks in their work, but they seem to be a minority. So it seems especially important for mentors of female students, postdocs, and young faculty, to provide encouragement in this regard. This same issue may apply to other minorities in science as well.

### 4. Enjoy your work!

It is quite easy in today's science to get caught up in the “external rewards” game, meaning: seeking praise, high profile publications, and honors or awards. But these are transient and illusory rewards. The prestigious prizes and high profile publications are often a lottery—in addition to some of the factors above, there is a lot of luck involved in who happens upon the “really big” discoveries. One may or may not get lucky, and may or may not get recognition for that. Sometimes recognition only comes after the prime of one's career—John Fenn received the Nobel Prize at 85 years old. That's a long time to wait for reward if you're just doing science for the sake of such rewards (I doubt that was Fenn's motivation for discovering electrospray ionization).

A different and much more gratifying way to pursue a career is to simply enjoy the work! Do science for the sake of doing it. This is as likely as anything to lead to big discoveries and fame. But even if those things don't happen, you are enjoying yourself, and life is too short not to do so.

### 5. Learn to say “No!”

Over the span of a career, one gets asked to do many non-science activities: serving on committees, grant reviews, paper reviews, and so on. While it is important to contribute effort to these things to keep the system functioning, it is necessary to set a limit, so that they don't take over the fun of doing science itself. The system will not collapse just because one says “no” from time to time in order to preserve time to do science. Learning to say “no” is particularly important for young faculty, who find themselves barraged with such requests, and who can easily get sucked into full-time committee duties. It is wise to step back frequently and ask, “overall, is this work I am doing fun?” If the answer is no, perhaps it is time to revisit points 1 and 4 above, and consider diving into a new area.

### 6. Learn to enjoy the process of writing and presenting.

Note the distinction in this guideline from: “learn to write and present well.” Many students I encounter dislike writing more than anything else they do. As a result, when it comes time to write a paper, it is a struggle from start to finish, both for them and for those working with them. When one doesn't like doing something, procrastination is the most common response. Procrastination and good writing don't mix. I say this even though I am someone who, as an undergraduate, would work all night on a term paper to turn it in at the last moment, and often receive an “A”. But in the real world of scientific paper writing, that first draft just won't cut it. It usually takes three or more significant rewritings and lots of input from others to get it right. Combine that with procrastination and it's a recipe for not getting a good paper out in a timely fashion, or perhaps not at all.

So the key is to figure out how to enjoy the writing process, thereby encouraging oneself to avoid procrastination. There is no one formula that works for everyone—some people need utter peace and quiet for their writing. Others prefer writing at a coffee shop, or to have music playing. The thing is to figure out what works, and to stick with it, training oneself to have positive mental associations with writing.

Robert Boice, in his book *Advice for New Faculty Members*, suggests the key is to do a little bit of writing every day [4]. The goal is simply get the ideas on the page, without worrying about their form at the beginning. By doing this a little bit every day—perhaps only 30–60 minutes—it is amazing how quickly and enjoyably a big writing project can take shape through a process of gradual evolution.

This often takes significant retraining, however. Many of us begin with the notion that writing should come in sudden bursts of dramatic creation. This message is conveyed frequently in movies that portray an author writing a novel in a sudden last minute rush, and it is reinforced in high school and college by many of us learning to get away with writing papers at the last minute (and still doing well). Reprogramming that unrealistic expectation out of one's head is therefore a key to learning to enjoy writing.

The same principle applies to giving a good presentation: enjoy its making and giving. Forget everything you ever learned about giving dry, stuffy presentations (i.e., all those things in the document *How to Make a Scientific Lecture Unbearable*) [5]. While it is critical to have good science in your talk, it is equally critical to bring that science to life for the audience. That is nigh impossible if you are scared to death of being in front of the audience, or if you are completely bored by your subject matter. If you are bored, the audience will surely be bored, and you might as well not have wasted their time—or your own.

The last thing a reader or talk attendee wants to see is a bunch of data just to prove that you did some work. It is much more interesting to tell a story. The story begins with why you started the work in the first place (the big reasons, not just “because my advisor told me to”), it usually has mystery and intrigue (e.g., dead ends, which are worth reporting only if they helped lead you to the final answer), and some kind of dramatic conclusion (which challenges the audience to think about things in a new way). This may seem like overstatement, but having sat through many extraordinarily dry, boring scientific talks (and having read many dry papers), I find that the ones that stand out are those that have such elements. If there is a lack of enthusiasm for the work you are doing, that may be a sign that it's the wrong work for you to be doing.

It can be a fun challenge to figure out who your audience is and what they will respond to. For example, when I was a postdoctoral researcher, I once gave a group meeting presentation accompanied by sound effects borrowed from Monty Python. We all had a good laugh, and I still managed to convey some science, too. But I would never do this at a scientific conference. Yet at a conference with a series of 15 minute talks, it is still possible to give a presentation that stands out—by enjoying its making and giving, and fine-tuning it for that audience. Elements such as presenting clear, understandable slides, and providing adequate introduction and background to the audience are very important. But it is most important to discuss subject matter that you have enthusiasm about.

Once one has learned to enjoy writing and presenting, it is very likely that writing well and presenting well will follow, since it is more difficult to do a truly poor job of something one enjoys doing.

### 7. See the big picture and keep it in mind.

Understanding and conveying the big picture for one's work is perhaps the greatest challenge facing young scientists. It is difficult to make the transition from a life of undergraduate classwork—where every step is prescribed by the instructor—to the pursuit of authentic research in graduate school, where there is no a simple formula to follow to pursue a successful line of research. At the start of a research career, the subject matter is often prescribed by one's advisor, and as a result, it is very common for students to simply rely on the advisor's word that it is important work to be doing, without really thinking about it, in keeping with the earlier mode of operation from undergraduate days. This lack of introspection regarding the “why” translates into many problems down the road, including: bad presentations (because no motivation for the work is given), bad manuscripts (because no motivation for the work is given), and, often, bad morale (because one comes to feel like a robot turning a crank).

From the start, it is critical to be very familiar with the Why. Why are you doing the work? Who will care about it, either now, or in the future? Is it likely to have any benefit? Note that the answers to these questions are often not easy. Many times discoveries are made long before they are ever put to practical use, and that use is often well outside the vision of the originator. So the key to this point is to think about the Why, even if there is no simple answer. Another way of stating this is that there should be some explicit and stated motivation for the work, even if it is just “intellectual curiosity.” That kind of introspection will help with one's own motivation in doing the work, and just as importantly, this will translate into better presentations and papers (because of making it more fun, as discussed in point 6 above).

It would be gratifying to come up with guidelines 8–10 just for numerical conformity. However, lacking an additional and meaningful guideline to give, instead I would restate one in particular: guideline 4, enjoy your work. All of the great scientists I have encountered are those who really enjoy what they are doing.

The astute reader may notice that most of the above rules are about process, rather than end result. This is to counter a phenomenon endemic to our culture: results count, and so advice is usually tailored to how to get those results in the quickest and most obvious manner. However, by attempting to short-circuit the thinking about process, in order to achieve the quickest result, often the end result is not a better one, and more importantly, leads to little long-term gratification.

An example is the advice to “work hard.” While one who works hard is usually more productive than one who doesn't, working too hard can be counterproductive. The rule could instead be stated “work hard enough,” but then the question becomes: how much work is “hard enough”? That leads to a quagmire of endless debate about how much work might make one most productive (and even how said productivity is measured—is it citations, prestigious prizes, grant money, salary, or ...?).

If one focuses instead on the processes involved in doing science, then the answers to such questions are much more obvious. Enough work is exactly the amount at which one can maintain enjoyment of the process of work, without burning out (which is not enjoyable) or becoming socially isolated (which is not enjoyable). If that amount of work is not enough to maintain a scientific career, then a different career may need to be considered, where such enjoyment can be found. Because, in the end, one may have many medals or honors bestowed, but those are transient scraps of paper or metal. True satisfaction with doing something worthwhile lasts for a lifetime. 
